# From osmolytes to oxidative stress: Temporal exometabolite shifts and microbial community responses in rehydrated biocrusts

**DOI:** 10.1016/j.isci.2026.118091

**Published:** 2026-11-04

**Authors:** Teeratat Kaewjon, Nicole Pietrasiak, Paul De Ley, Jason E. Stajich, Nuttapon Pombubpa

**Affiliations:** 1 Department of Microbiology, Faculty of Science, Chulalongkorn University, Bangkok, Thailand; 2 School of Life Sciences, University of Nevada, Las Vegas, NV 89154, USA; 3 Department of Nematology, University of California-Riverside, Riverside, CA 92521, USA; 4 Department of Microbiology and Plant Pathology, University of California-Riverside, Riverside, CA 92521, USA

**Keywords:** drylands, biocrust, wetness duration, exometabolite, network, Mojave desert

## Abstract

Biocrusts are vital to dryland ecosystems, yet their microbe-exometabolite associations during rehydration remain poorly understood. This study investigated both bacterial and fungal diversity and exometabolite dynamics in light cyanobacterial crusts (LCCs) and cyanolichen crusts (CLCs) under rewetting over 96 h. Wetting increased the abundance of Firmicutes and reduced that of Actinobacteria within a day, while Shannon diversity remained stable in LCCs but declined in CLCs. Fungal communities were dominated by Ascomycota, with the lowest diversity at 24 h in LCCs. Targeted exometabolomics identified extracellular compounds categorized into three temporal clusters: early accumulation of amino acids, sugars, and osmolytes; intermediate divergence between LCCs and CLCs; and late enrichment of oxidative-stress-related metabolites. Network and Zi-Pi topology analyses identified urocanic acid, pipecolic acid, and S-adenosylhomocysteine as putative keystone metabolites, suggesting roles in microenvironmental regulation and microbial associations. These findings provide insights into the fundamentals of the resilience of biocrust communities during rewetting.

## Highlights

• Wetting increased Firmicutes and reduced Actinobacteria within 24 h
• Exometabolites showed initial, intermediate, and late response signatures
• LCCs showed fewer bacteria-fungi connections at the intermediate response than CLCs
• Urocanic acid, pipecolic acid, and SAH act as putative keystone metabolites

## Graphical abstract

**Figure:**
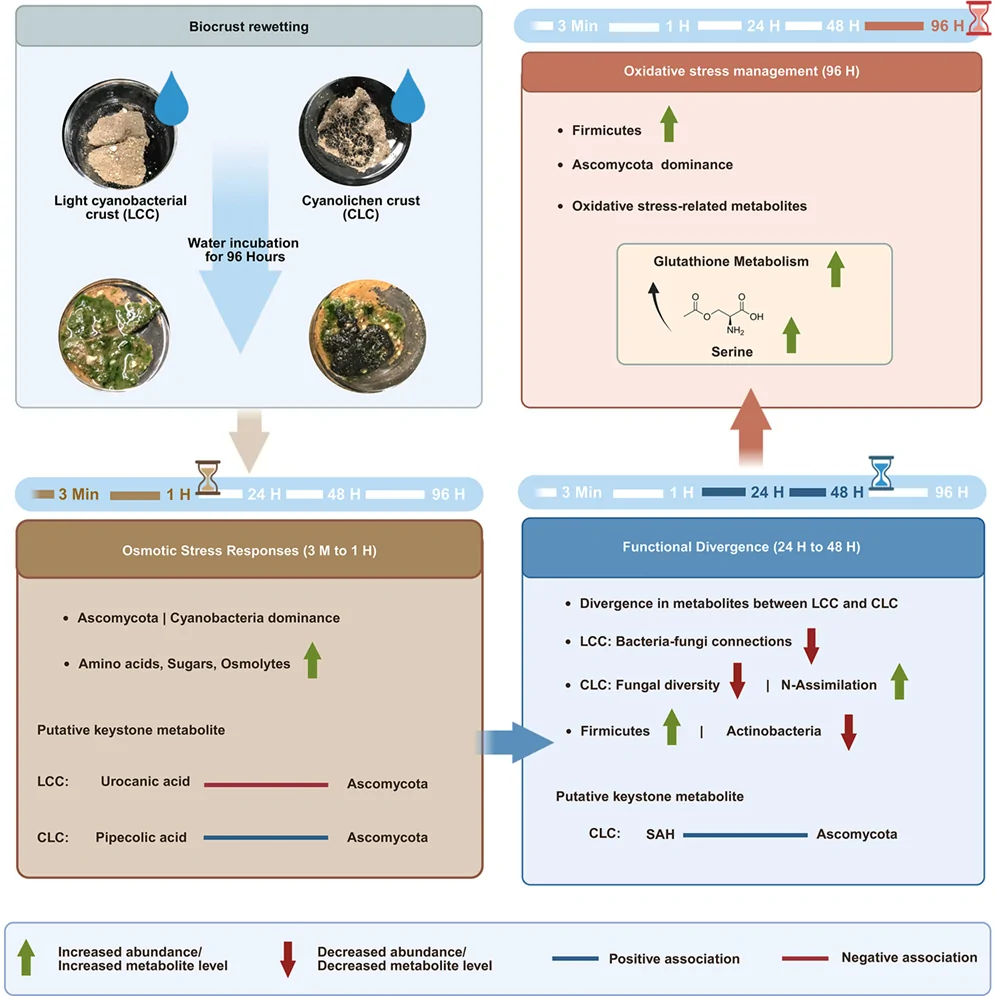

## Teaser

Biological sciences; Microbiology; Cell biology

## Introduction

Biological soil crusts (biocrusts), soil surface aggregates comprising mosses, lichens, cyanobacteria, eukaryotic algae, and other associated microbes, are well documented for their importance in terrestrial ecosystems. Through the adaptation of microbial activities in response to environmental changes, they release small molecules or metabolites into their surroundings, which play key roles in nutrient cycling, interspecies communication, and defense mechanisms.^1^^,^^2^ Unfortunately, climate change and land-use intensity could lead to a reduction in global biocrust coverage by more than 25% within a century.^3^ While prolonged droughts represent a hazardous consequence of climate change, biocrust survival hinges on the efficiency of their metabolic recovery during intermittent rainfall, making the investigation of post-wetness temporal dynamics critical to understanding their long-term resilience.

Biocrust classification has been developed based on a combination of physical characteristics, cohesiveness, and the abundance of photosynthetic organisms. In desert landscapes undergoing primary or secondary succession, biocrust types can serve as indicators of biocrust development, following a predictable sequence of community changes based on local environmental conditions.^4^ In general, unconsolidated soil is initially colonized by filamentous cyanobacteria, which bind soil particles to form a thin layer on the soil surface called “cyanobacterial crust.” Once filamentous cyanobacteria have stabilized the soil and microclimatic conditions are favorable, lichens and moss crusts can develop, with complex symbioses between heterotrophic microorganisms (bacteria and fungi) and photobionts (usually cyanobacteria, microalgae, and bryophytes) forming natural associations.^3^ Additionally, differences in the establishment, abundance, and distribution of biocrust types in the landscape can also indicate micro- and patch-scale landscape heterogeneity linked to differences in microclimate and soil properties.^5^^,^^6^^,^^7^^,^^8^

Advances in technologies for profiling environmental samples, especially through the use of amplicon sequencing, help determine the composition of the microbial community and provide qualitative clues about functional potential within biocrusts. Studies show that biocrust development tracks changes in bacterial and fungal communitiesin terms of taxonomic composition and diversity.^9^^,^^10^ The structure of microbial communities depends on the adaptability of their species to certain ecological niches and nutrient limitations. Previous studies in the Negev Desert have shown that certain microbial taxa are associated with wetting-triggered successional processes, indicating that local environmental conditions shape biocrust communities.^5^ These findings highlight the importance of investigating multiple sites, regions, and crust types to better understand spatial variations in microbial profiles. Equally importantly, to observe and understand how microbial communities respond to changing conditions, particular crust types must be analyzed repeatedly over time.^2^^,^^11^

While amplicon sequencing has significantly advanced our understanding of microbial composition, it is less suited for quantifying functional aspects and microbial activity. Desert biocrusts remain dormant to withstand stressors such as temperature fluctuations and desiccation; thus, their nutrient cycling functions are highly episodic.^12^^,^^13^ Precipitation events initiate hydration, and the duration of surface wetness is one of many factors governing biocrust development, as it triggers the transition of most biocrust organisms from the dormant stage.^2^^,^^11^^,^^14^ These responses involve shifts in microbial communities, transcriptional patterns, and concentrations of various small molecules in the soils within hours and days after rehydration.^2^^,^^14^^,^^15^ According to Swenson et al.,^2^ after initial wetting, cyanobacterial crusts primarily release fatty acids, which gradually dissipate over time, whereas prolonged wetness results in the release of most amino acids and nucleobases. This difference may indicate changes in metabolite consumption and substrate preferences, as well as shifts in community structure. Direct investigation of how microbes alter metabolite concentrations in their surroundings is possible using exometabolomic techniques, which reveal substrate utilization and potential metabolic interactions.^2^^,^^16^ However, such investigations of dynamics in biocrusts remain limited. To fill this knowledge gap, changes in the abundances of community microorganisms and metabolites during biocrust reawakening induced by water activation can improve models of species interactions. This will clarify not only how the microbes in different biocrust types respond to wetting but also how they cooperate or compete to sustain their biocrust community.

In this study, we examine changes in microbial community composition after biocrust water activation using amplicon sequencing and capture biocrust exometabolite profiles to identify intracellular microbial metabolites that may be released into biocrust soil water. Our specific objectives include (i) investigating the microbial community members and their exometabolites involved in the biocrust reactivation during the successional process triggered by a single wetting event and (ii) identifying putative keystone taxa with inferred exometabolic functions potentially linked to community reawakening. We conducted these studies using two types of biocrusts commonly found at Joshua Tree National Park in the Mojave Desert, including light cyanobacterial crusts (LCCs) and cyanolichen crusts (CLCs). Both biocrust types exhibit distinct morphological forms. We expected (i) microbial communities to rapidly recover activity following the single pulse wetting event and to undergo subsequent shifts in community composition over the post-wetting time series; and (ii) keystone species to synthesize distinct metabolites for different biocrust types.

## Results

### Temporal shifts in bacterial and fungal taxa during biocrust wetting

Single-pulse wetting strongly altered microbial diversity and community structure throughout the 96-h incubation. Beta diversity showed clear temporal shifts in bacterial communities in both LCCs (Permutational Multivariate Analysis of Varience [PERMANOVA], *p* = 0.001, *R*^2^ = 0.535) and CLCs (PERMANOVA, *p* = 0.001, *R*^2^ = 0.418) biocrusts (Figures 1A and 1B). Shannon index values revealed biocrust-specific responses. The Shannon index of the LCC remained relatively stable (Analysis of Varience [ANOVA], *F*(4,19) = 2.123, *p* = 0.118) (Figure 1C). In contrast, the CLC exhibited significant temporal changes (ANOVA, *F*(4,20) = 5.653, *p* = 0.003), with the lowest values at 48 h (Figure 1D).

**Figure 1:**
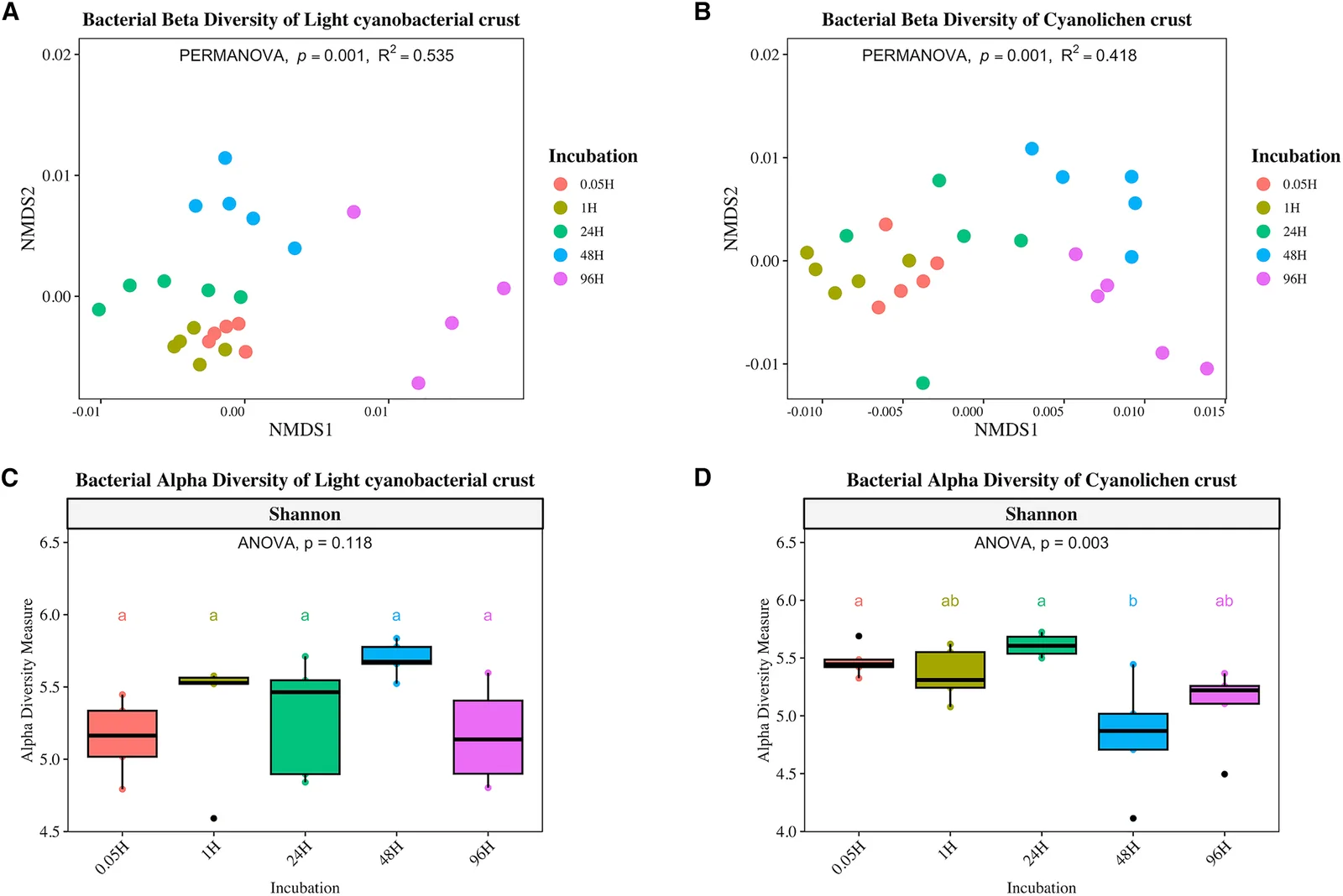
Temporal changes in bacterial diversity of two biocrust types during incubation (A) Beta diversity of the light cyanobacterial crust. (B) Beta diversity of the cyanolichen crust. (C) Shannon diversity of the light cyanobacterial crust. (D) Shannon diversity of the cyanolichen crust. Boxplots show the 25th and 75th percentiles, while the median is shown as a line inside the box. Error bars show the 1st and 99th percentiles. Different letters indicate significant differences among incubation times based on Tukey’s Honestly Significant Difference (HSD) test (*p* < 0.05). Overall differences among incubation times were assessed using ANOVA for Shannon diversity and PERMANOVA for beta diversity, with significant results (*p* < 0.05) indicated in the corresponding panels.

Fungal communities shown in the Non-metric Multidimensional Scaling (NMDS) plot exhibited significant temporal restructuring (Figure 2). Shannon index values in LCCs decreased at 24 h and increased by 96 h (*F*(4,20) = 3.201, *p* = 0.0348) (Figure 2C), whereas Shannon index values in CLCs progressively declined (*F*(4,20) = 4.076, *p* = 0.0141) (Figure 2D).

**Figure 2:**
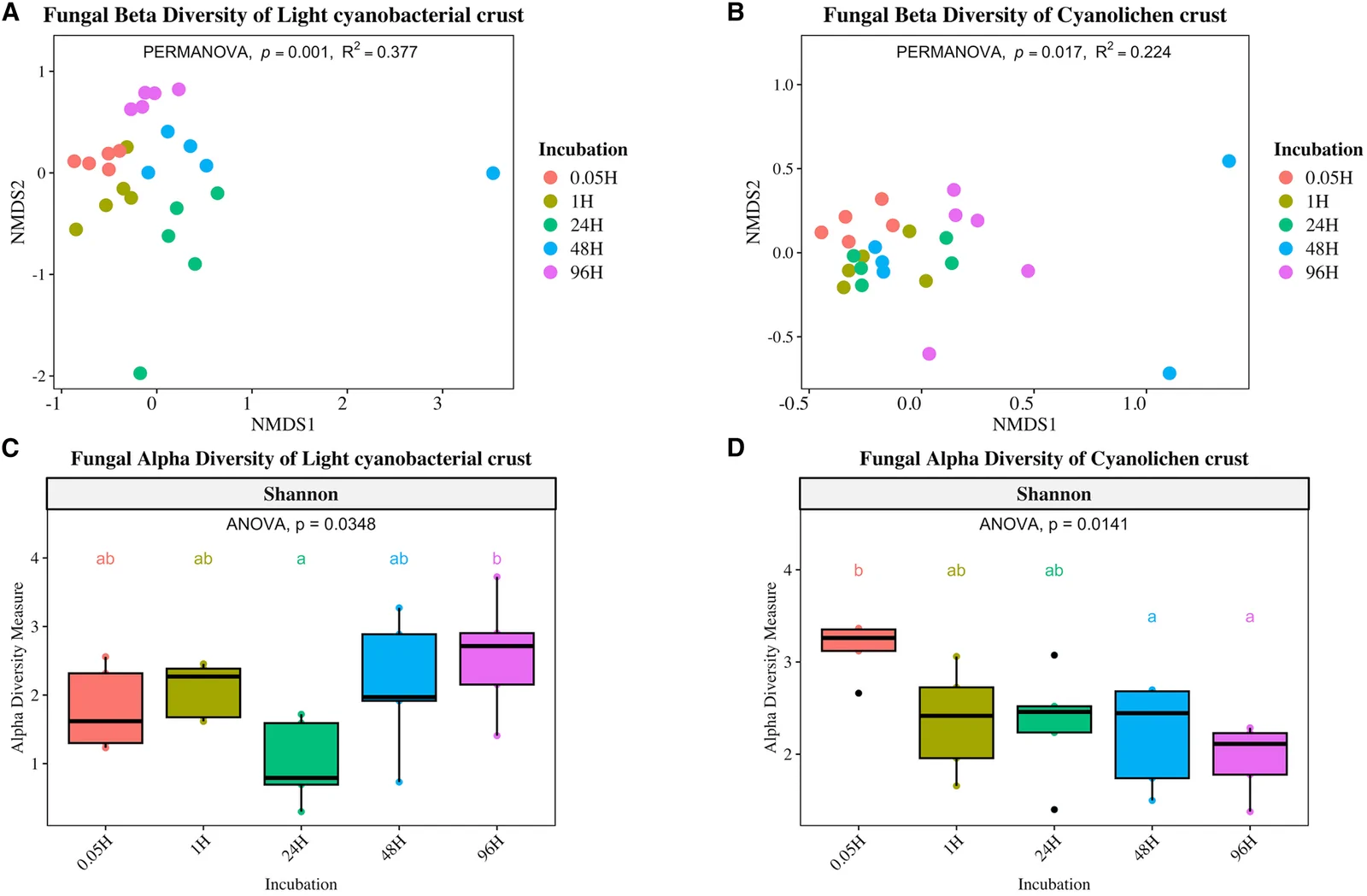
Temporal changes in the fungal diversity of two biocrust types during incubation (A) Beta diversity of the light cyanobacterial crust. (B) Beta diversity of the cyanolichen crust. (C) Shannon diversity of the light cyanobacterial crust. (D) Shannon diversity of the cyanolichen crust. Boxplots show the 25th and 75th percentiles, while the median is shown as a line inside the box. Error bars show the 1st and 99th percentiles. Different letters indicate significant differences among incubation times based on Tukey’s HSD test (*p* < 0.05). Overall differences among incubation times were assessed using PERMANOVA for beta diversity and ANOVA for Shannon diversity, with significant results (*p* < 0.05) indicated in the corresponding panels.

Bacterial community composition exhibited consistent patterns of change over a long time series after wetting events. Cyanobacteria in LCCs decreased significantly in relative abundance from 79.3% to 44.0%, while those in Firmicutes increased from 0.1% to 15.9%, becoming predominant after 48 h, compared to 3 min (0.05 h) (Figure 3A; Table S1). On the other hand, in CLC samples, the phyla Cyanobacteria (24.9–39.9%) and Proteobacteria (27.4–34.8%) were dominant across wetting durations. Notably, the relative abundance of Actinobacteria began to decline after the initial 24-h wetness in both crust types. In fungal communities, Ascomycota exhibited particularly high relative abundance in CLCs (85.2–97.6%). Basidiomycota constituted 1.5–10.8%, with the highest relative abundance in the LCC after 24 h of wetness (Figure 3B; Table S1).

**Figure 3:**
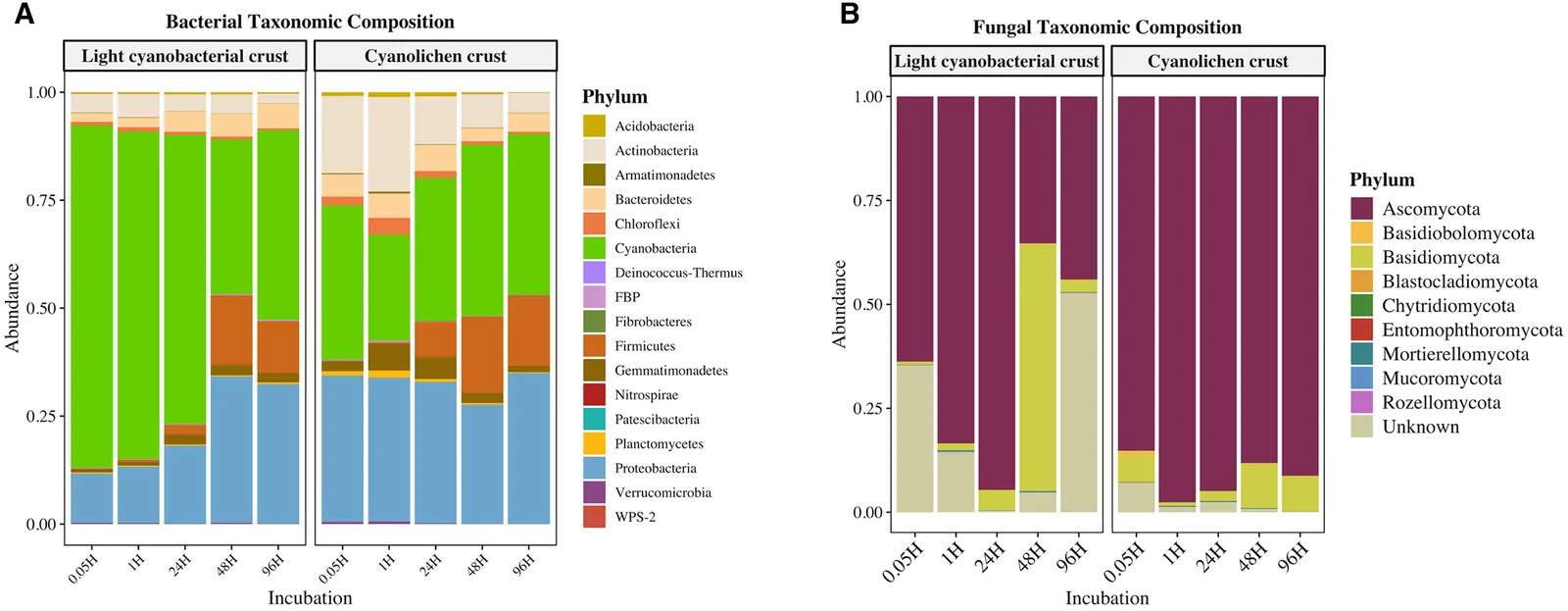
Taxonomic composition of bacterial and fungal communities in two biocrust types (A) Bacterial phylum composition of the light cyanobacterial crust and cyanolichen crust. (B) Fungal phylum composition of the light cyanobacterial crust and cyanolichen crust.

Analysis of the fungal community’s ecological functions using FUNGuild (Figure S1) indicated that lichenized fungi dominated both biocrust types (52.1–81.7%), while ectomycorrhizal fungi (EMF) exhibited contrasting trajectories. LCC samples showed a peak at 24 h, while CLC samples demonstrated a gradual increase through 96 h. In addition, saprotrophic fungi increased moderately in LCCs at later time points.

### Shifts in exometabolite patterns after wetting in different biocrust types

A total of 50 known metabolites were identified in the samples and represented on the principal component analysis (PCA) plot (Figure 4). The result clearly illustrated distinct patterns in the exometabolite composition of LCC and CLC samples. These changes were allocated to three classes, including initial responses (3 min–1 h), intermediate responses (24–48 h), and late responses (96 h), which were statistically evaluated using PERMANOVA (*R*^2^ = 0.830, *p* = 0.001).

**Figure 4:**
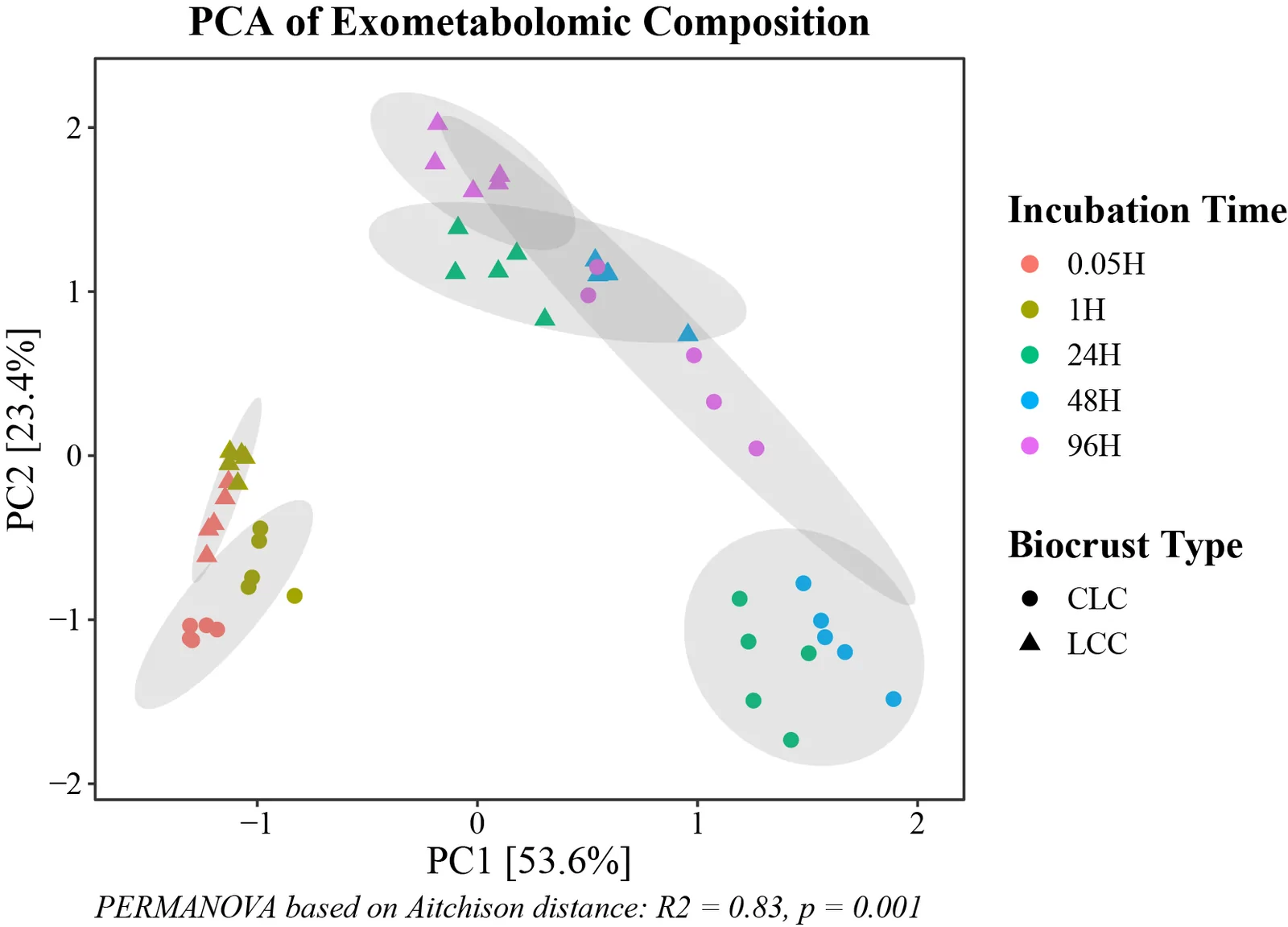
Principal component analysis of exometabolomic compositional trajectories The ordination, based on center log-ratio-transformed metabolite abundances, illustrates distinct shifts in metabolic profiles over 96 h of rewetting. Colors represent incubation time points (0.05–96 h), while shapes distinguish between LCC and CLC biocrust types. 95% confidence ellipses are shown for each time point to highlight community grouping.

During the intermediate phase (24–48 h), PCA showed a clear separation between LCC and CLC exometabolite profiles. Pathway analysis showed that LCC samples were preliminarily enriched in nitrogen-related pathways, including “alanine, aspartate, and glutamate metabolism,” “arginine metabolism,” and “arginine biosynthesis” during this phase (Figure S2). These pathways corresponded with high concentrations of N-rich intermediates such as asparagine, arginine, and ornithine (Figure S3). Conversely, CLC samples at this phase showed an enrichment in “pyrimidine metabolism,” “beta-alanine metabolism,” “pantothenate and Coenzyme A (CoA) biosynthesis,” “lysine degradation,” “biotin metabolism,” and “valine, leucine, and isoleucine biosynthesis,” supported by increased levels of 4-guanidinobutyric acid, carnitine, lysine, and threonine. In the late phase (96 h), both crust types exhibited high levels of intermediate involvement of “glutathione metabolism,” with increased levels of serine.

### Microbial-exometabolite relationships, putative keystone taxa, and exometabolites under the reawakening process

Three metabolites from CLC samples exhibited temporal patterns that were not different from those of water control samples at each time point (Data S1). These metabolites were excluded from the network analysis. The top 500 amplicon sequence variant (ASV) counts from each fungal and bacterial dataset were selected for network analysis to integrate microbiomics and exometabolomics. Six bacterial-fungal-metabolite association networks under biocrust types and metabolic response clusters were constructed. The linkages refer to positive or negative correlations (Figures 5 and 6). Remarkably, the number of connections between bacteria and fungi in co-occurrence varied, particularly during the intermediate and late stages when comparing the two crust types (Table S2). The number of bacteria-fungi connections in LCC samples dropped from 793 at the initial phase to 66 connections at the intermediate phase. In contrast, the number of connections between fungi and bacteria doubled during the intermediate stages of CLC compared with the initial stage, although few interspecies links were observed during the late response.

**Figure 5:**
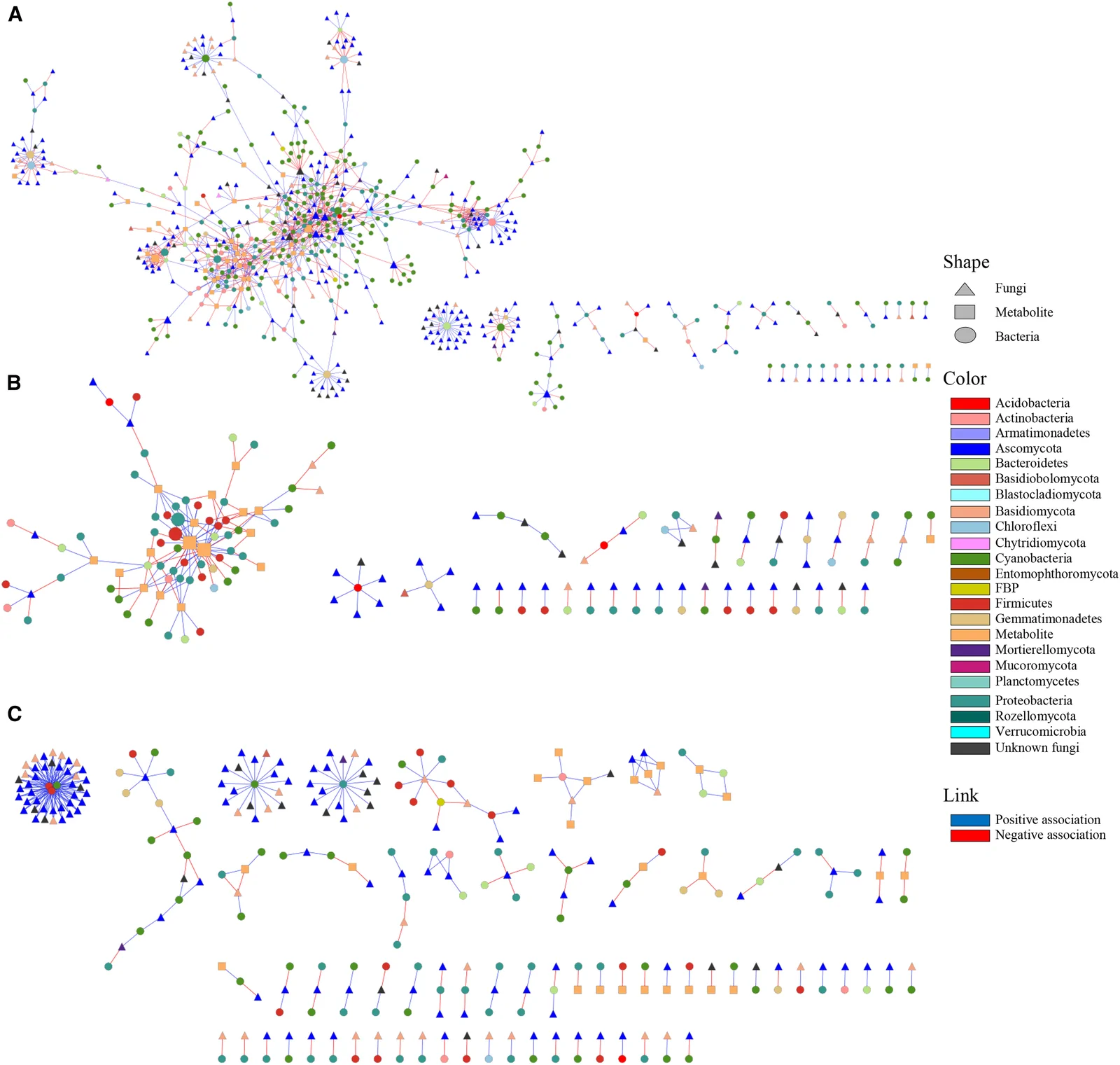
Co-occurrence network analysis and Zi-Pi plot of LCC crust type under three response clusters (A) Initial response cluster. (B) Intermediate response cluster. (C) Late response clusters. Network nodes represent individual ASVs and exometabolites. ASVs are distinguished by shape and color according to their kingdom and phylum, whereas exometabolites are shown as separate node types. Edges indicate significant positive (blue) and negative (red) associations between nodes.

**Figure 6:**
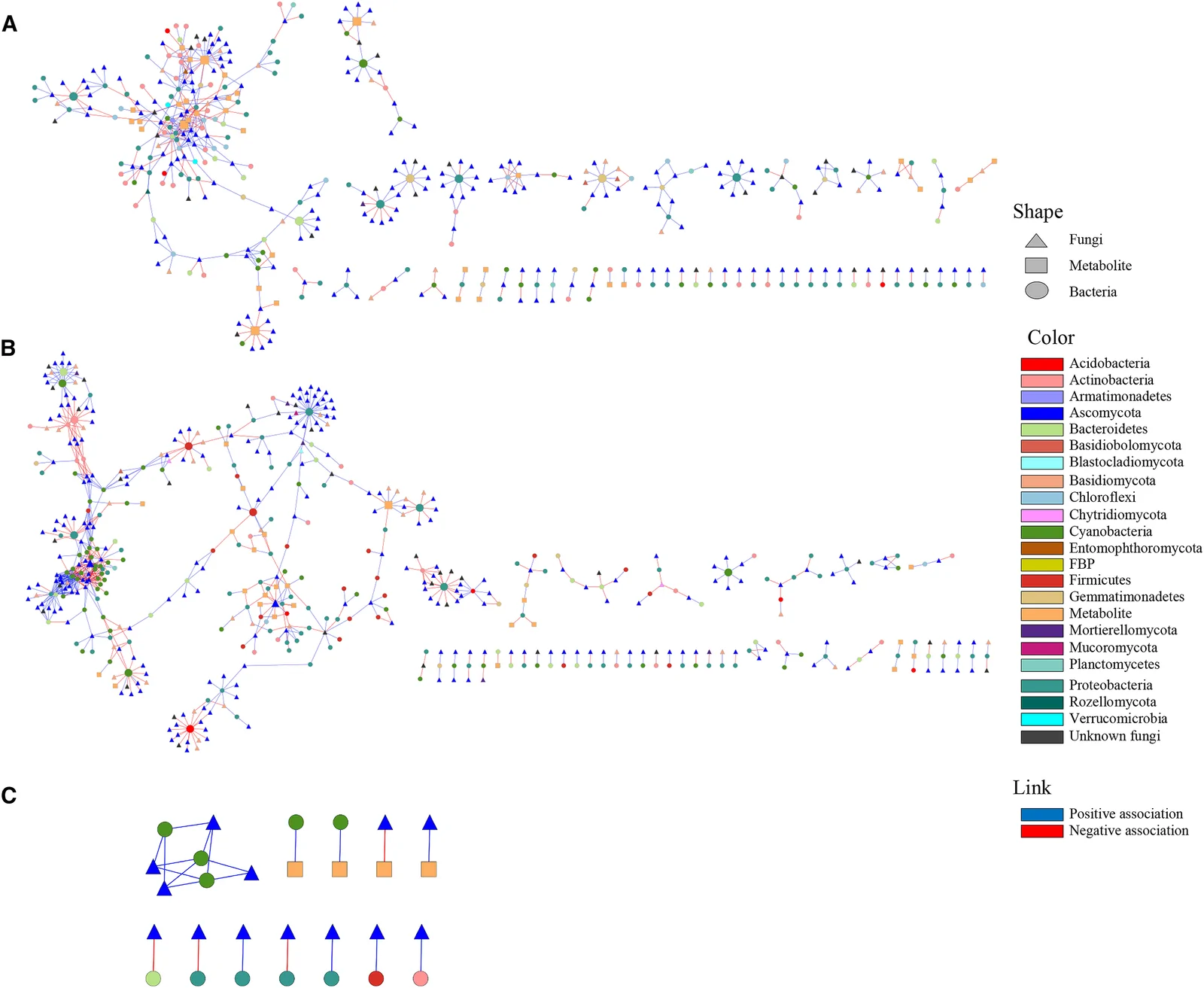
Co-occurrence network analysis and Zi-Pi plot of CLC crust type under three response clusters (A) Initial response cluster. (B) Intermediate response cluster. (C) Late response clusters. Network nodes represent individual ASVs and exometabolites. ASVs are distinguished by shape and color according to their kingdom and phylum, whereas exometabolites are shown as separate node types. Edges indicate positive (blue) and negative (red) associations between nodes.

Zi-Pi analysis identified putative keystone taxa and exometabolites within the two biocrust types. In the LCC samples, 35 putative keystone nodes were identified (12 from fungi, 19 from bacteria, and 3 exometabolites) (Table S3). Additionally, in the CLC samples, 26 keystone nodes were identified (20 from Bacteria, 2 from Fungi, and 4 exometabolites) (Table S4). In the LCC sample at the initial response cluster, Fungi_ASV176, classified as *Coniothyrium telephii*, a plant pathogen/wood saprotroph, was identified as a module hub in module 5 (Figure S4A). Fungi_ASV176 showed a negative relationship with Cyanobacteria within its module. In the late-response cluster of LCC samples, Fungi_ASV380 was classified as *Filobasidium magnum*, an undefined saprotroph, and identified as a module hub in module 3 (Figure S4B). Fungi_ASV380 mainly displayed positive associations with Firmicutes within its module. The putative bacterial keystones were represented, including Proteobacteria, Bacteroidetes, Gemmatimonadetes, and various members of Actinobacteria (Table S3).

These taxonomic keystone taxa co-occurred with specific putative keystone exometabolites that varied by crust type and response clusters. At the initial response in LCC samples, urocanic acid emerged as a module hub, exhibiting positive associations with Proteobacteria (Bacteria_ASV372) and negative associations with Ascomycota (Figure S5). Conversely, pipecolic acid was identified as a putative keystone exometabolite in CLC samples, showing positive associations with Ascomycota. During the intermediate response cluster, 5-aminovaleric acid and 3-ureidopropionic acid were identified and had positive associations with Proteobacteria and Firmicutes. In contrast to CLC samples, S-adenosylhomocysteine (SAH) was identified as a putative keystone exometabolite that had positive associations with Ascomycota.

## Discussion

### Microbial succession following a single wetting event differs between biocrust types

Microbial communities in biocrusts respond rapidly to local environmental conditions, especially moisture exposure, which triggers shifts in composition and diversity.^6^ Our investigation of biocrust reawakening processes monitored microbial dynamics across 96-h wetness periods. Our results support findings from field measurements in the Negev Desert, which indicated that surface wetness duration most strongly determined biocrust biomass and biocrust type and that it also strongly influenced bacterial and fungal composition.^5^^,^^6^^,^^17^

Biocrust types respond differently in their immediate changes to microbial composition and diversity following a single wetting event. Our results showed that the Shannon index values of LCC samples did not change significantly (Figure 1A), indicating stable alpha diversity overall. Notable taxonomic shifts nevertheless occurred in community composition. During the first 24 h, Cyanobacteria were dominant, while Firmicutes showed a significant increase. Heterotrophic bacteria, such as Actinobacteria in CLC samples, were dominant in the early stages of wetting but decreased when Firmicutes bloomed at 24 h. Our findings were consistent with several studies reporting Firmicutes blooming in biocrusts soon after rewetting.^1^^,^^2^^,^^11^ Firmicutes have been characterized as early responders to moisture in biocrusts and soils because many members form desiccation-resistant endospores that rapidly germinate when water and soluble nutrients become available.^2^^,^^15^ In addition, Actinobacteria are enriched in mature biocrust types (e.g., lichen and moss crusts) compared with other biocrust types.^18^^,^^19^ This relative abundance of Actinobacteria decreased upon rewetting, however, which could indicate a competitive disadvantage during the first hours of wetness.

Fungal communities displayed varying patterns in both alpha and beta diversity. The alpha diversity (Shannon) of the biocrust LCC decreased 24 h after wetting but then increased significantly by 96 h, suggesting that perhaps dormant taxa emerged in the period. On the other hand, alpha diversity in the biocrust CLC decreased significantly and fairly steadily over time, suggesting a successional process favoring a subset of fungal taxa. Taxonomic composition was dominated by the phylum Ascomycota, followed by Basidiomycota in both crust types. Our results are consistent with research in deserts at the Colorado Plateau, the Mu Us Sandy Land, and Oman.^20^^,^^21^ This sustained dominance of Ascomycota in crusts is likely attributed to the high melanin content in their cell walls, a physiological adaptation that provides vital protection against extreme abiotic stressors, such as prolonged desiccation and intense UV radiation.^20^^,^^21^

### Exometabolites reflect metabolic activity: Specific patterns of metabolic pathways within biocrust types following wetting

Our study revealed dynamic changes in the composition of exometabolites (Figure 4). In particular, samples taken at 3 min and 1 h were most similar to each other, while being most distinct from those taken after other wetting periods. The most frequently identified metabolites during the first hour after water addition included amino acids, amino sugars, purines, pyrimidines, and osmolytes (such as betaine) in both crust water types. These metabolites presumably correspond to cellular damage and metabolite leakage due to rewetting events,^2^^,^^22^ as well as the release of metabolites through mechanosensitive channels during osmotic shifts^23^^,^^24^ and non-catastrophic micro-ruptures in cell walls and membranes that are subsequently repaired.^25^ Rapid rewetting of the soil forces microbes to eliminate accumulated osmolytes, either by respiring or transporting molecules across the cell membrane, to prevent excessive water from entering cells and causing catastrophic cell wall rupture.^26^ This explains the elevated presence of such as betaine in the crust soil water.

During the intermediate response, defined as the 24- to 48-h wetness periods, we noted a marked change in the composition of exometabolites. This alteration distinctly separated the samples of the CLC group, which clustered together, from those of the LCC group. The divergence was further clarified by pathway enrichment analysis, revealing specific metabolic pathways active in each sample type. In LCCs, the primary metabolic theme identified was rapid protein synthesis and nitrogen assimilation, as evidenced by enrichment in amino acid and arginine pathways. These processes are likely driven by the increased activity of EMF. EMFs are recognized for their ability to utilize nitrate as a sole nitrogen source, thereby enhancing nitrogen uptake and facilitating its transfer from soil to host plants.^27^ Nitrate importer genes are consistently found in EMF genomes.^28^^,^^29^ Consequently, we observed high concentrations of nitrogen-rich compounds (asparagine, arginine, and ornithine) and an increase in EMF abundance. The accumulation of nitrogen-rich compounds in the crust water solution suggests low microbial nitrogen use efficiency during this phase. According to Mooshammer et al.,^30^ the rapid nitrogen assimilation by microbes likely exceeds their available carbon supply in this phase. The microbial community prioritizes meeting carbon demands, resulting in the partitioning of acquired organic N toward excretion rather than toward biomass stabilization, thereby forming a transient pool of organic N. In contrast, CLC samples displayed a highly active and integrated metabolism encompassing crucial processes, including DNA and RNA biosynthesis, synthesis of a key precursor to CoA, and energy production. These active pathways were characterized by increases in 4-guanidinobutyric acid, carnitine, and threonine.

Subsequently, the late-response cluster, identified after 96 h h of wetness in both LCC and CLC samples, was characterized by further shifts in exometabolite composition under prolonged wetness. In this phase, we observed the continuation of stress-response metabolic pathways in the fungal system, particularly an increase in glutathione metabolism, which was linked to elevated levels of serine found in the biocrust soil water. This suggests that fungi may be facilitating glutathione cycles to detoxify reactive oxygen species,^30^ a reaction that has been previously documented in fungi such as *Aspergillus* species under oxidative stress.^31^

### Putative keystone exometabolite and its associations among microbial communities

Our integrative network analysis of the microbiomes and exometabolomes of two biocrust types, LCC and CLC, demonstrated different patterns of microbial dynamic shifts. Network analysis revealed distinct patterns of inter-kingdom linkages, particularly during the intermediate response phase (24–48 h post-rewetting). In LCC samples, the number of bacterial-fungal connections decreased markedly from 793 at the initial to 66 linkages at the intermediate response cluster. In contrast, CLC samples exhibited a sharp increase from 365 to 729 linkages, reflecting a highly integrated and presumably cooperative phase of microbial associations. Consistent with the observed metabolite composition, this phase exhibited compositional divergence, reaching a separation far greater than that observed at the initial wetting (3 min) or later stage (96 h). These contrasting association dynamics during the intermediate phase likely influence the composition of extracellular metabolite pools in soil water, as enhanced bacterial and fungal connectivity is associated with greater nutrient mobilization and metabolic efficiency.^32^

A keystone species is a key player that has a disproportionately large influence on the structure and function of a community relative to their abundance. The presence or lack of these species could result in considerable cascading impacts across the network, possibly changing the overall structure and functioning of the ecosystem.^33^^,^^34^ In this study, the topological roles of nodes in a co-occurrence network and Zi-Pi plot analysis were used to identify putative keystone taxa and the most influential exometabolites. We focused on exometabolites identified as putative keystone metabolites, as these compounds may play pivotal roles in mediating microbial interactions by maintaining community stability or facilitating resilience.

In the initial LCC response cluster, urocanic acid emerged as a module hub, negatively associated with Ascomycota. The presence of an imidazole ring within its structure is noteworthy, as this motif is associated with potent antifungal activity and the disruption of mitochondrial membrane potential.^35^^,^^36^ Although our results do not demonstrate a direct antifungal mechanism, the negative association between urocanic acid and Ascomycota suggests that urocanic acid may contribute to the suppression of early fungal activity during wetting. On the other hand, pipecolic acid emerged as a putative keystone metabolite in the same time frame in CLC samples, exhibiting a positive association with Ascomycota. Pipecolic acid is an intermediate in the biosynthesis of penicillin, compounds with well-documented antibacterial activity.^37^ Its positive association with Ascomycota may reflect metabolic processes related to fungal activity or competitive microbial interactions during early wetting.

In the intermediate response cluster of LCC samples, SAH was identified as a putative keystone exometabolite at this time frame. SAH is a metabolic intermediate that is converted through the activated methyl cycle into precursors of autoinducer-2 (AI-2).^38^ Through the LuxS/AI-2 system, AI-2 functions as a quorum-sensing signal involved in bacterial communication and has also been implicated in bacterial-fungal interactions.^39^ Therefore, the presence of SAH may reflect an increased potential for AI-2-related signaling, which could contribute to the bacterial-fungal associations observed in CLC samples.

### Microbial dynamics during biocrust activation and its implication in biocrust management

Biocrust activation of is a rapid process driven by hydration and is essential for the functioning and health of dryland ecosystems. For effective biocrust management, it is crucial to understand and facilitate these activation dynamics to maintain soil health, prevent erosion, and preserve ecosystem services in arid environments.

Efforts to restore biocrusts are being advanced through innovative cyanobacteria inoculation technologies, which can serve as initial colonizers in natural biocrust ecosystems.^40^^,^^41^ We identified putative key fungi, bacteria, and exometabolites involved in the establishment of microbial networks. We identified certain heterotrophic bacteria, such as those in the phyla Proteobacteria (e.g., *Rubellimicrobium* sp., *Microvirga* sp.), Bacteroidetes (*Adhaeribacter* sp.), Gemmatimonadetes (*Longimicrobium* sp.), and Actinobacteria, as putative keystone bacteria in community networks. Nelson et al.^42^ revealed that adding beneficial heterotrophic bacteria to the cyanobacterial consortium increases phototrophic biomass yields in biocrust inoculum. The increase in biomass yields is primarily due to a symbiotic division of labor within the cyanosphere, in which heterotrophic bacteria provide essential fixed nitrogen to the cyanobacteria in exchange for photosynthesized carbohydrates. Additionally, we identified *F. magnum* (Fungi_ASV380) as a putative keystone fungus. Despite its low abundance (Figure S6), it stands out as a candidate due to its “module hub” status and its positive association with Firmicutes during the late response of LCC biocrusts. *F. magnum*, a basidiomycetous yeast, produces extracellular polymeric substances that enhance its tolerance to environmental stress and contribute to improved soil structural stability.^43^ In fungal-bacterial co-cultivation, *F. magnum* produces oxidative enzymes that transform complex organic compounds, whereas *Bacillus* species secrete hydrolytic enzymes that release simpler nutrients from proteins and polysaccharides.^44^ Their complementary metabolic functions may promote mutual benefits. This suggests that *F. magnum* might play a crucial role in promoting the structure and function of biocrust communities in prolonged wetting. However, fungal probiotics for biocrust inoculum have not been explored to date.

Conversely, fungi showing negative associations with desired pioneer taxa (e.g., Cyanobacteria suppressors, such as *C. telephii* in initial LCCs and *Thyrostroma cornicola* in intermediate CLCs) should be carefully evaluated and potentially excluded from initial inoculation consortia. Rather than a one-size-fits-all approach, consortia should be tailored to the local biocrust community assembly and the environmental conditions of the restoration site, e.g., by identifying adapted local biocrust types, their microniches, and the microclimatic conditions likely required. Additionally, putative keystone exometabolites were identified in different response clusters. The selection of metabolites for the biocrust inoculum should be carefully considered, as metabolites play a key role in shaping ecological niches and thus may determine which microbes are better able to develop than others.

In conclusion, microbial communities and metabolic networks in biocrusts undergo substantial restructuring in response to wetting and moisture-driven activation, with distinct trajectories observed between LCC and CLC types. Bacterial communities showed shifts in the composition and abundance of Cyanobacteria and Firmicutes. These shifts reflect different responses during the successional process triggered by wetting, with early bacterial responders such as Firmicutes emerging more rapidly, whereas changes in fungal communities appeared to proceed more gradually under prolonged wetness. Exometabolite patterns reflected distinct stages across the time series, ranging from osmotic stress responses to oxidative stress management. Co-occurrence networks revealed putative keystone metabolites, including urocanic acid, pipecolic acid, and SAH. These emerged as critical modulators of microbial associations, perhaps influencing both competitive and cooperative dynamics. Functional roles were inferred from association patterns, offering a foundational framework for future experimental validation of keystone taxa and metabolite-mediated interactions in biocrust systems. Such approaches can open pathways to enhance soil health and ecosystem resilience in arid landscapes facing altered precipitation regimes.

### Limitations of the study

This study was conducted under laboratory conditions using sterile LC/MS-grade water and constant incubation time to observe microbial community changes and wetting-triggered metabolite production. While this experimental design reduced environmental variability and enabled reproducible temporal comparisons, it may not fully capture the complexity of natural wetting events, in which fluctuating temperature, light, and rainfall intensity can influence microbial responses and extracellular metabolite profiles.^2^^,^^15^ In addition, the microbiome-exometabolome relationships identified through the SPIEC-EASI network inference and Zi-Pi topology analyses represent statistical associations rather than direct ecological interactions. As a result, future experimental validation through controlled microcosm experiments will be necessary to establish their causal roles in structuring biocrust microbial communities during rehydration.^11^^,^^44^

## Resource availability

### Lead contact

Requests for further information and resources should be directed to and will be fulfilled by the lead contact, Nuttapon Pombubpa (nuttapon.po@chula.ac.th).

### Materials availability

This study did not generate new unique reagents. All materials used are commercially available or previously published.

### Data and code availability

• The sequencing data generated in this study have been deposited in the NCBI Bioproject (GenBank: PRJNA1401472).
• The unprocessed mass spectrometry data in this study have been deposited in the MassIVE repository (MassIVE: MSV000103359; https://doi.org/10.25345/C5N010798).
• The metabolomic data summary generated in this study has been deposited in Zenodo (Zenodo: 22790320; https://doi.org/10.5281/zenodo.22790320).
• All custom codes for statistical analysis and visualization in this study are available in the GitHub repository (https://github.com/natpombubpa-lab/Biocrust_Reawakening).
• Additional information: Any additional information required to reanalyze the data reported in this paper is available from the lead contact upon request.

## Acknowledgments

This research was supported by the 90th Anniversary of 10.13039/501100002873Chulalongkorn University Scholarship under the Ratchadapisek Somphot Endowment Fund. This work was partially supported by the Office of the Permanent Secretary, Ministry of Higher Education, Science, Research and Innovation (TSRI; grant no. RGNS 65-005). We thank Joshua Tree National Park (Permit JOTR-2017-SCI-0010), the Mojave National Preserve (MNP; Permit MOJA-2015-SCI-0017), the University of California Natural Reserve System, and the Sweeney Granite Mountains Desert Research Center (SGNDRC) for permission to conduct research (https://doi.org/10.21973/N3S942). We thank Robert Lee Graduate Student Research Grants from the Joshua Tree National Park Association awarded to N.P. and N.P. Fungal ITS and bacterial 16S primer sequences and barcodes were provided by the Alfred P. Sloan Foundation Indoor Microbiome Project. Support for J.S. was provided through the 10.13039/100005825USDA National Institute of Food and Agriculture Hatch project CA-R-PPA-211-5062-H CIFAR, where J.S. is a fellow in the Fungal Kingdom: Threats and Opportunities program. Data analyses were performed on the High-Performance Computing Cluster at the University of California, Riverside, in the Institute of Integrative Genome Biology, supported by the 10.13039/100000001National Science Foundation (grant nos. DBI-1429826, DBI-2215705) and the 10.13039/100000002National Institutes of Health (grant no. S10-OD016290).

## Author contributions

Conceptualization, T.K.; methodology, T.K., N.Pi., P.D.L., and N. Po.; investigation and data curation, N. Pi., P.D.L. and J.E.S.; formal analysis, T.K.; writing – original draft, T.K.; writing – review and editing, N.Pi., P.D.L., J.E.S., N.Po.; supervision, N.Po.; project administration, N.Po.; funding acquisition, N.Po. All authors discussed results and approved the final manuscript.

## Declaration of interests

The authors declare that they have no known competing financial interests or personal relationships that could have appeared to influence the work reported in this paper.

## STAR★Methods

### Key resources table

**Table:** 

REAGENT or RESOURCE	SOURCE	IDENTIFIER
**Biological samples**		

Light cyanobacterial crust (LCC) and cyanolichen crust (CLC)	Joshua Tree National Park in the Mojave Desert^9^	N/A

**Chemicals, peptides, and recombinant proteins**		

QIAGEN DNeasy PowerSoil kit	Qiagen	Germantown, MD, USA

**Deposited data**		

Metabarcode sequencing	NCBI BioProject	GenBank: PRJNA1401472 (https://www.ncbi.nlm.nih.gov/bioproject/PRJNA1401472/)
Raw metabolomic data	MassIVE	MassIVE: MSV000103359 (https://doi.org/10.25345/C5N010798)
Metabolomic data summary	Zenodo	Zenodo: 22790320 (https://doi.org/10.5281/zenodo.22790320)
All custom code	GitHub	https://github.com/natpombubpa-lab/Biocrust_Reawakening

**Software and algorithms**		

QIIME2 v2019.1	Bolyen et al.^45^	https://doi.org/10.1038/s41587-019-0209-9.
DADA2	Callahan et al.^46^	https://doi.org/10.1038/nmeth.3869.
AMPtk pipeline	Palmer et al.^47^	https://doi.org/10.7717/peerj.4925.
R software	R foundation	https://www.r-project.org/
phyloseq R package	McMurdie et al.^48^	https://doi.org/10.1371/journal.pone.0061217
MetaboAnalyst 6.0	Pang et al.^49^	https://doi.org/10.1093/nar/gkae253.
SPIEC-EASI R package	Kurtz et al.^50^	https://doi.org/10.1371/journal.pcbi.1004226
Cytoscape software	Shannon et al.^51^	https://doi.org/10.1101/gr.1239303

### Method details

#### Sampling sites and biocrust sample collections

Biocrust samples were collected from Joshua Tree National Park (GPS: 34.10 N, −115.45 W) in the Mojave Desert as part of previous research.^9^ Briefly, light cyanobacterial crust (LCC) and cyanolichen crust (CLC, dominated with *Collema* spp.) were collected using a spatula and sterile sampling technique, with 5 replicates per crust type. Biocrust samples were stored on ice and transported to a −80 °C freezer at the University of California, Riverside.

#### Experimental design

Five biological replicates were collected for each biocrust type. Biocrust incubation was done using 1 g of biocrust with 2 mL of sterile liquid chromatography–mass spectrometry (LC/MS)-grade water in 24-well plates (adapted from Swenson et al.^2^) and incubated for 96 h. Biocrust soil water was collected at 5 different time points (3 min, 1 h, 24, 48, and 96 h) with five replicates each. Water control samples (one replicate of LC/MS-grade water, no soil) was included and sampled at five time points to account for background signals originating from the water. At each time point, 2 mL of biocrust soil water was collected directly from the well and transferred to sterile tubes. The collected soil water was centrifuged at 5000×*g* for 5 min and the supernatant was used for LC-MS analysis. Supernatant was placed in new 2 mL tubes and stored at −80 °C for metabolite profiling. Metabolite profiling of biocrust soil water samples was conducted using liquid chromatography–mass spectrometry (LC-MS) at the Metabolomics Core Facility, University of California, Riverside. Compound detection and identification were performed using the facility’s in-house metabolite database. LC-MS/MS metabolomics was performed on a Xevo TQ-XS triple quadrupole mass spectrometer (Waters) coupled to an ACQUITY UPLC I-Class FTN system (Waters). Separations used a SeQuant ZIC-pHILIC 5 μm polymeric (150 × 2.1 mm; EMD Millipore Corporation, 1504600001) column held at 50 °C, with water (A) and acetonitrile (B) at 200 μL/min and the following gradient: 0 min, 90% B; 1.5 min, 90% B; 16 min, 20% B; 18 min, 1% B; 22 min, 1% B; 23 min, 90% B; 33 min, 90% B. Samples injection volumes were 1 μL. Electrospray ionization source conditions were as follows: capillary voltage was 1.0 kV in positive mode and 2.0 kV in negative mode; desolvation gas was set to 1100 L/h; cone gas was set to 150 L/h; nebulizer gas was 6.7 bar; collision gas was argon (0.15 mL/min). Data were acquired in scheduled multiple reaction monitoring (MRM) mode with polarity switching. The method used 258 transitions for 132 metabolites. After water collection, the remaining biocrust soils in the well were processed through amplicon metabarcode sequencing, to examine microbial composition of different crust types after different incubation times.

#### DNA extraction and amplicon sequencing

The biocrust soils were subjected to DNA extraction using the QIAGEN DNeasy PowerSoil kit (Qiagen, Germantown, MD, USA) following the manufacturer’s protocol. The bacterial 16S rRNA gene (V4 region) and fungal ITS1 region were amplified as described in Pombubpa et al.,^9^ using the primer pairs 515F/806R for 16S and ITS1F/ITS2 for ITS1, employing the same thermocycling conditions, PCR cleanup, and library preparation for high-throughput sequencing. Amplicons were sequenced on an Illumina MiSeq platform using paired-end 2 × 250 bp reads.

The 16S V4 amplicon sequences were analyzed using QIIME2 16S processing workflows (v2019.1).^45^ Sequence reads were trimmed, followed by quality control filtering by DADA2.^46^ Filtered sequence reads were processed using DADA2 to generate 11,167 amplicon sequence variants (ASVs). Taxonomic assignment was carried out with the q2-feature-classifier plugin in QIIME2, utilizing SILVA reference database version 132.

Fungal ITS1 sequences were analyzed using the AMPtk pipeline.^47^ Paired-end reads were demultiplexed and reads with primer mismatches or lengths shorter than 100 bp were discarded. The reads were trimmed to set their length at 250 bp. Primer sequences were removed, and paired-end reads were merged with USEARCH (v9.2.64). Merged reads were quality filtered with an expected error threshold of 0.9^52^ and denoised, partitioning the unique reads into 1942 ASVs. Chimeric ASVs were discarded using VSEARCH (v2.13.6).^53^ Finally, the 1842 retained ASVs were taxonomically assigned using the UNITE database.^54^ In addition, mitochondria, chloroplasts, and singleton sequences were removed from the datasets, resulting in 6936 ASVs for bacteria and 1422 ASVs for fungi. Functional guilds were assigned using FUNGuild (v1.0).^55^ Both bacterial and fungal data were analyzed for diversity using the Phyloseq package^48^ in R version 4.4.2 and RStudio version 2024.12.1 + 563.

#### Data analysis

Levene’s test was used to analyze the homogeneity of variances of 16S rDNA and ITS gene region counts in the samples by ‘leveneTest’ function in the ‘car’ package. One-way analysis of variance (ANOVA) was performed using Tukey’s test (*p* < 0.05) for evaluating differences in alpha diversity with the ‘anova’ function and ‘TukeyHSD’ function. Beta diversity was compared employing Bray-Curtis distance with PERMANOVA via the ‘adonis’ function in the ‘vegan’ package for fungal data, while the weighted UniFrac distance was used for bacteria.

Raw chromatographic peak areas were screened against the LC/MS-grade water controls using a fold-change threshold calculated from the raw abundance data (Data S1) to identify and remove solvent-derived background metabolites prior to downstream analyses. Exometabolomic peak area quantitative data were Center Log-Ratio (CLR) transformed to account for data compositionality. Compositional trajectories were visualized using Principal Component Analysis (PCA) based on Euclidean distance and validated statistical significance of temporal shifts with PERMANOVA (999 permutations). Screened differential exometabolites were analyzed using MetaboAnalyst 6.0 for pathway identification (raw *p* < 0.05 and pathway impact >0.1), selecting the metabolic KEGG pathway (Kyoto Encyclopedia of Genes and Genomes) and *Saccharomyces cerevisiae* as a reference model.

Linkages between the microbiome and exometabolome were explored through network analyses with the ‘SPIEC-EASI’ package’s cross-domain interaction pipeline,^50^ and the network was visualized via Cytoscape software.^51^ We included only ITS ASV- Metabolite, 16S ASV-Metabolite, and ITS-16S ASV pairs to reduce compositional data bias from amplicon sequences and metabolite abundances.^33^ Putative keystone taxa and exometabolites were identified based on the different roles of the nodes (ASVs and exometabolites) in networks using within-module connectivity (Zi) and among-module connectivity (Pi) analysis. Zi-Pi analysis classified nodes into 4 categories, including (I) network hubs (Zi ≥ 2.5, Pi ≥ 0.62), (II) module hubs (Zi ≥ 2.5, Pi < 0.62), (III) connectors (Zi < 2.5, Pi ≥ 0.62), and (IV) peripherals (nodes connected in modules with few outside connections, Zi < 2.5, Pi < 0.62).^33^^,^^56^ Nodes with the important roles of module hubs, connectors, and network hubs in ecological networks were thus identified as putative keystone taxa and major exometabolites in this study.

### Quantification and statistical analysis

For each crust type, five independent biological replicates were collected at each incubation time point, including 3 min, 1 h, 24 h, 48 h, and 96 h per time point (*n* = 5). Differences in bacterial and fungal alpha diversity were evaluated using one-way ANOVA followed by Tukey’s HSD post hoc test. Microbial community differences were tested by PERMANOVA (999 permutations). Statistical significance was defined as *p* < 0.05. The SPIEC-EASI package was used to infer sparse associations among bacterial ASVs, fungal ASVs, and exometabolites based on compositional abundance data. Exact sample sizes and additional statistical details are provided in the corresponding figure legends and method details.

## Supplemental information

Document S1. Figures S1–S6 and Tables S1–S4

Data S1. Exometabolite fold change analysis
