# Supplementary material for: From osmolytes to oxidative stress: Temporal exometabolite shifts and microbial community responses in rehydrated biocrusts
Source: iScience. 2026 Nov 4;29(11):118091. doi: 10.1016/j.isci.2026.118091 (PMC13756169; doi:10.1016/j.isci.2026.118091)
Supplement: Supplementary file 1 [file mmc1.pdf]

iScience, Volume *29*

Supplemental information

From osmolytes to oxidative stress: Temporal exometabolite shifts and microbial community responses in rehydrated biocrusts

Teeratat Kaewjon, Nicole Pietrasiak, Paul De Ley, Jason E. Stajich, and Nuttapon Pombubpa

Supplementary figure

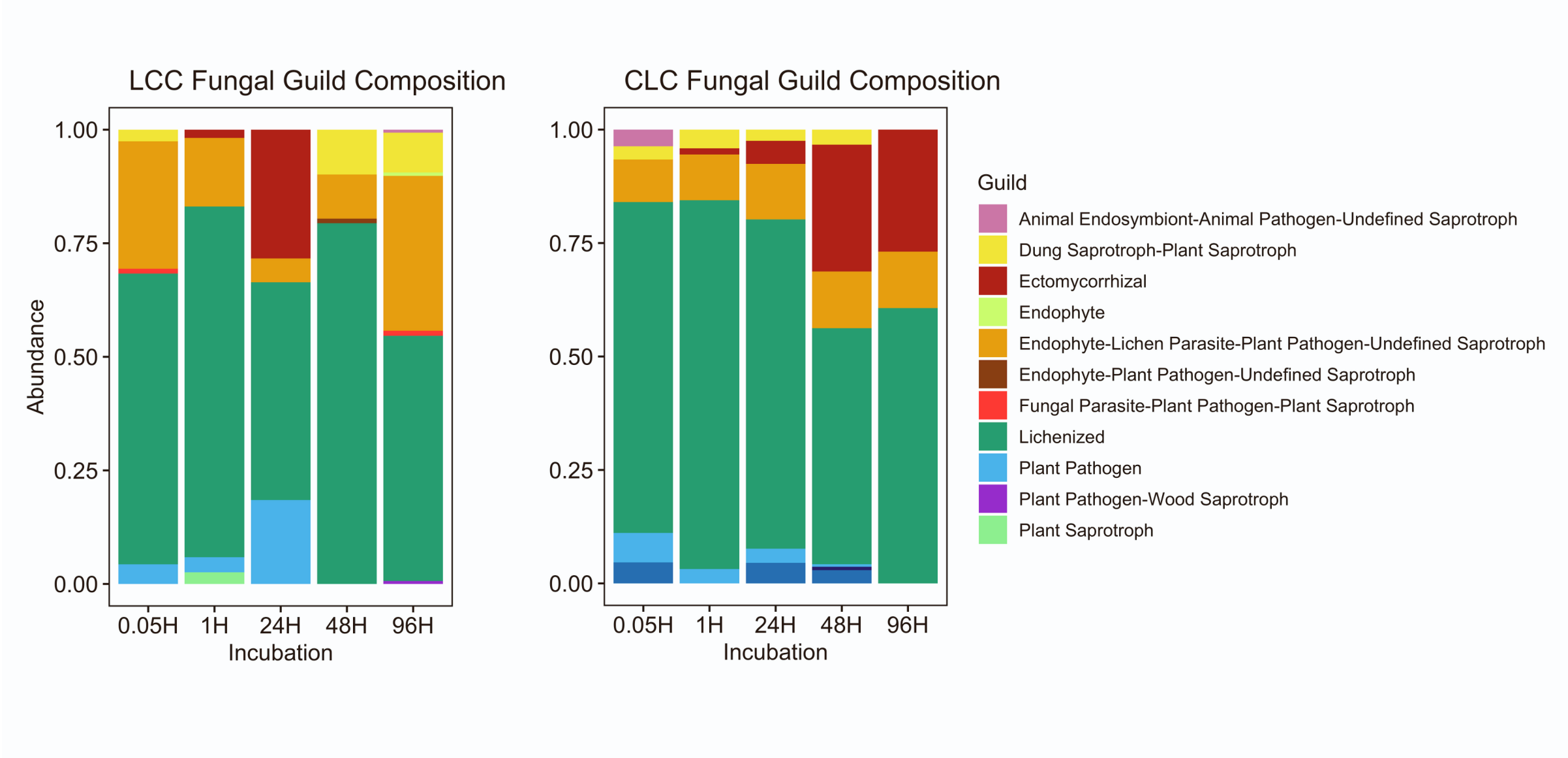

Figure S1. Guild composition of fungal communities of biocrust LCC and CLC.

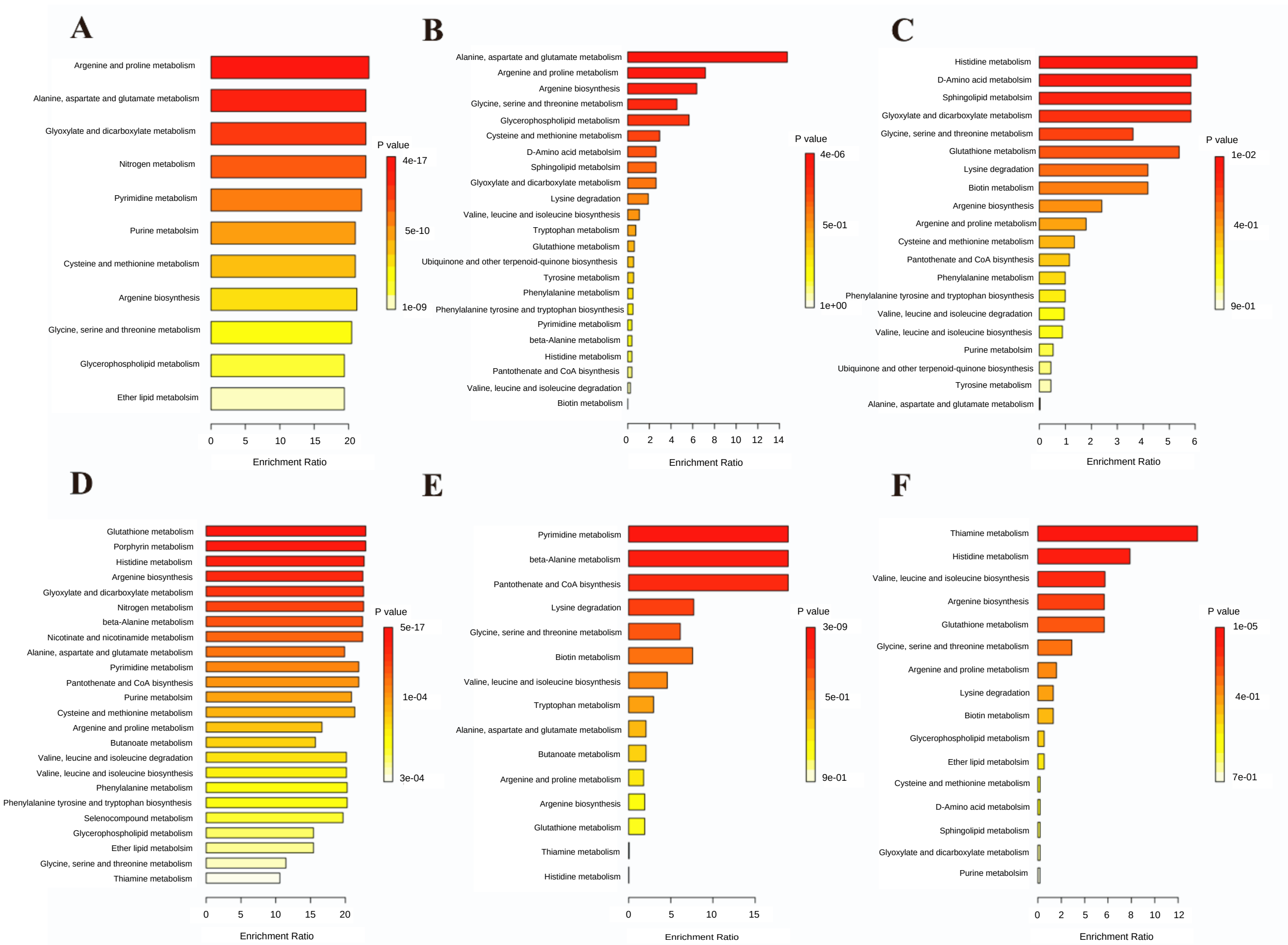

Figure S2. Pathway enrichment using differential exometabolite levels of three response clusters. Top pathways of LCC samples at the initial (A), intermediate (B), and late (C) response cluster, the initial (D), intermediate (E), and late (F) response clusters of CLC samples. The enrichment ratio (X-axis) represents the ratio of observed to expected metabolite hits in each pathway, and the *p*-value (color scale)

indicates the statistical significance of pathway enrichment, with lower *p*-values representing greater significance.

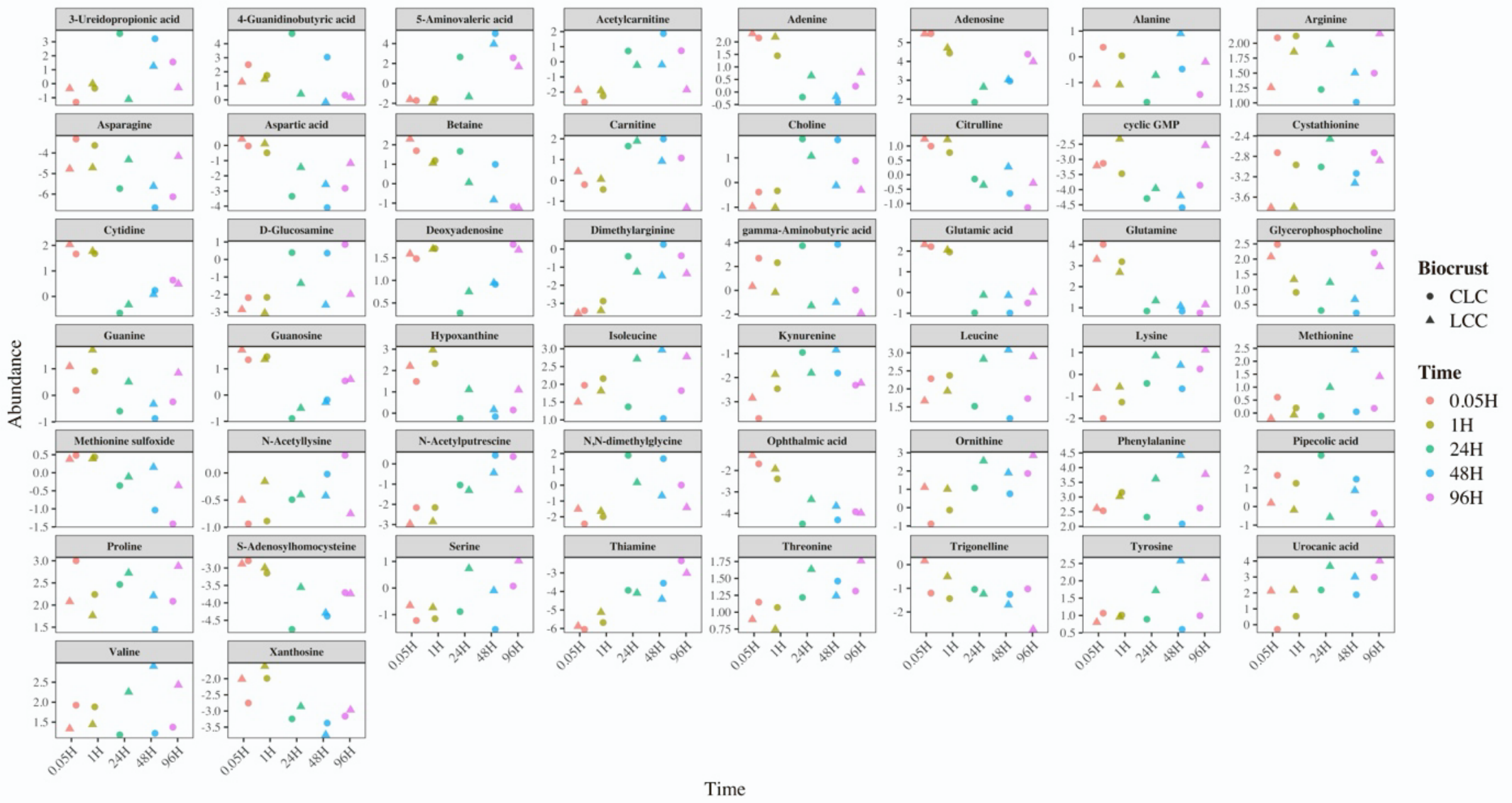

Figure S3. Metabolite level in the centered-log ratio transformation. Different colors represent different incubation times. Triangle and circle shapes represent biocrust type LCC and CLC, respectively.

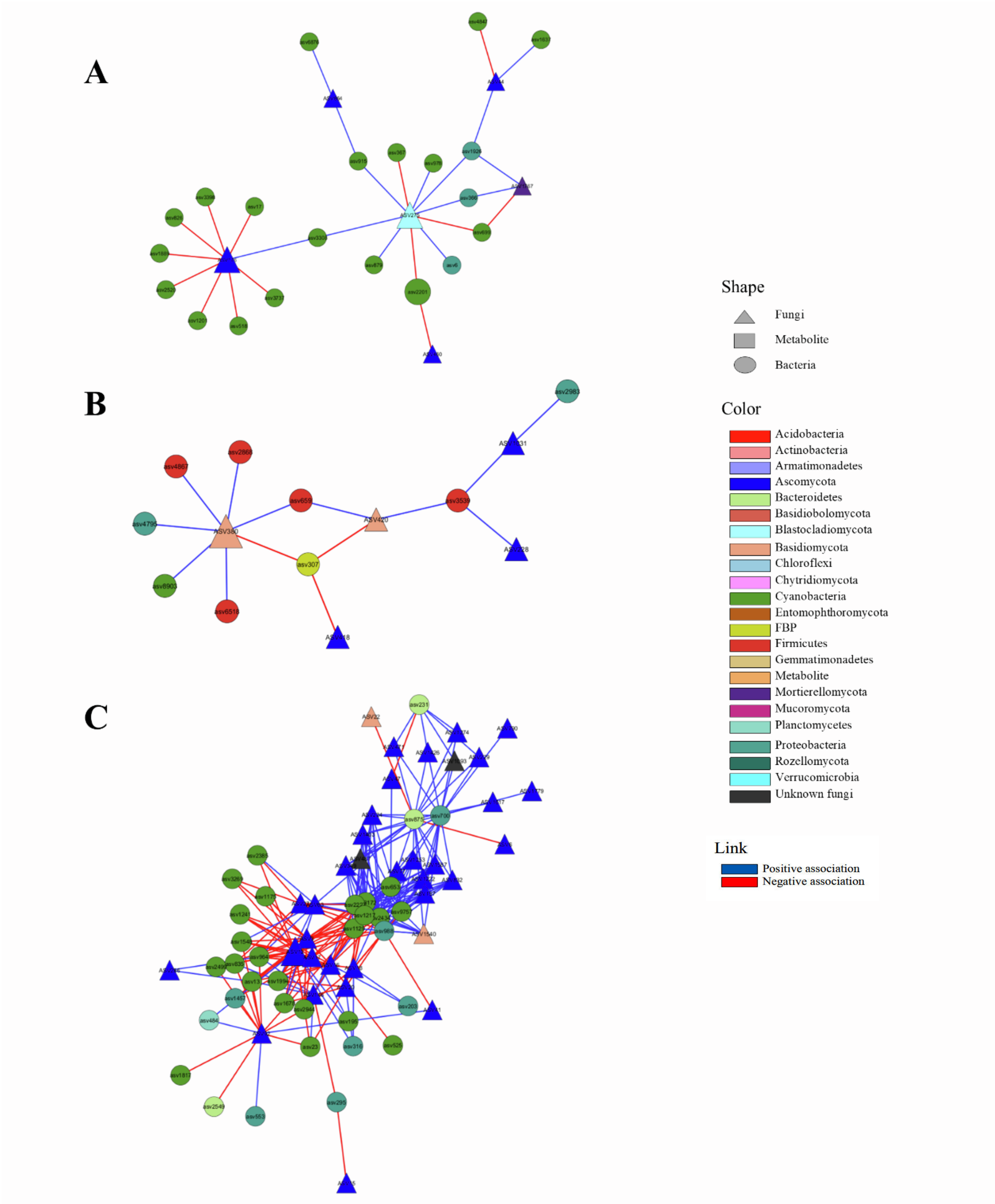

Figure S4. Examples of putative keystone bacteria and fungi identified at the initial response (A) and late response (B) of LCC samples, and intermediate response of CLC samples (C). Network nodes represent individual ASVs and exometabolites. ASVs are distinguished by shape and color according to their kingdom and phylum, whereas exometabolites are shown as separate node types. Edges indicate positive (blue) and negative (red) associations between nodes.

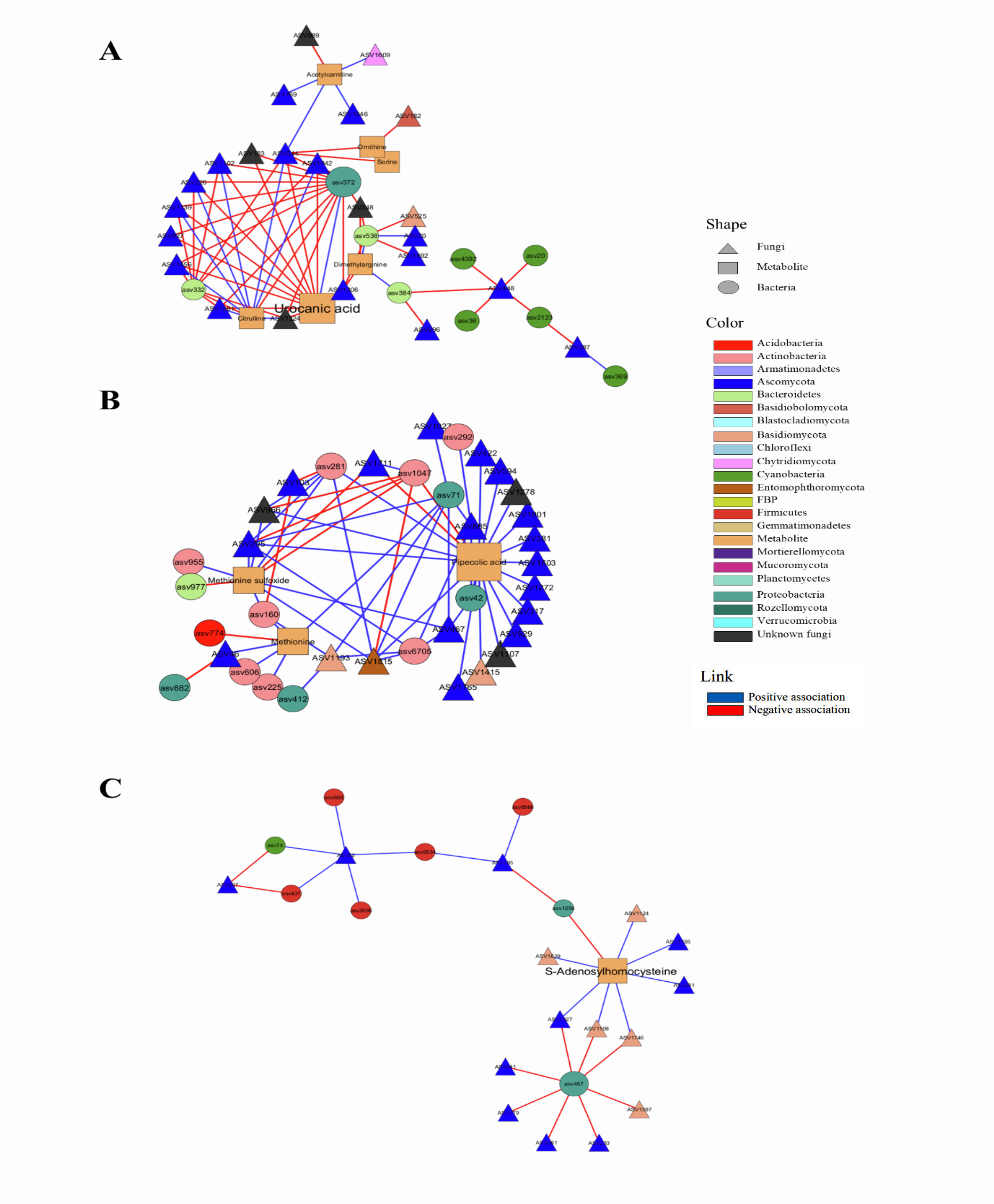

Figure S5. The putative keystone exometabolites that are mostly associated with fungi were identified at the initial response of LCC (A) and CLC (B), and the intermediate response of CLC samples (C). Network nodes represent individual ASVs and exometabolites. ASVs are distinguished by shape and color according to their kingdom and phylum, whereas exometabolites are shown as separate node types. Edges indicate positive (blue) and negative (red) correlations between nodes.

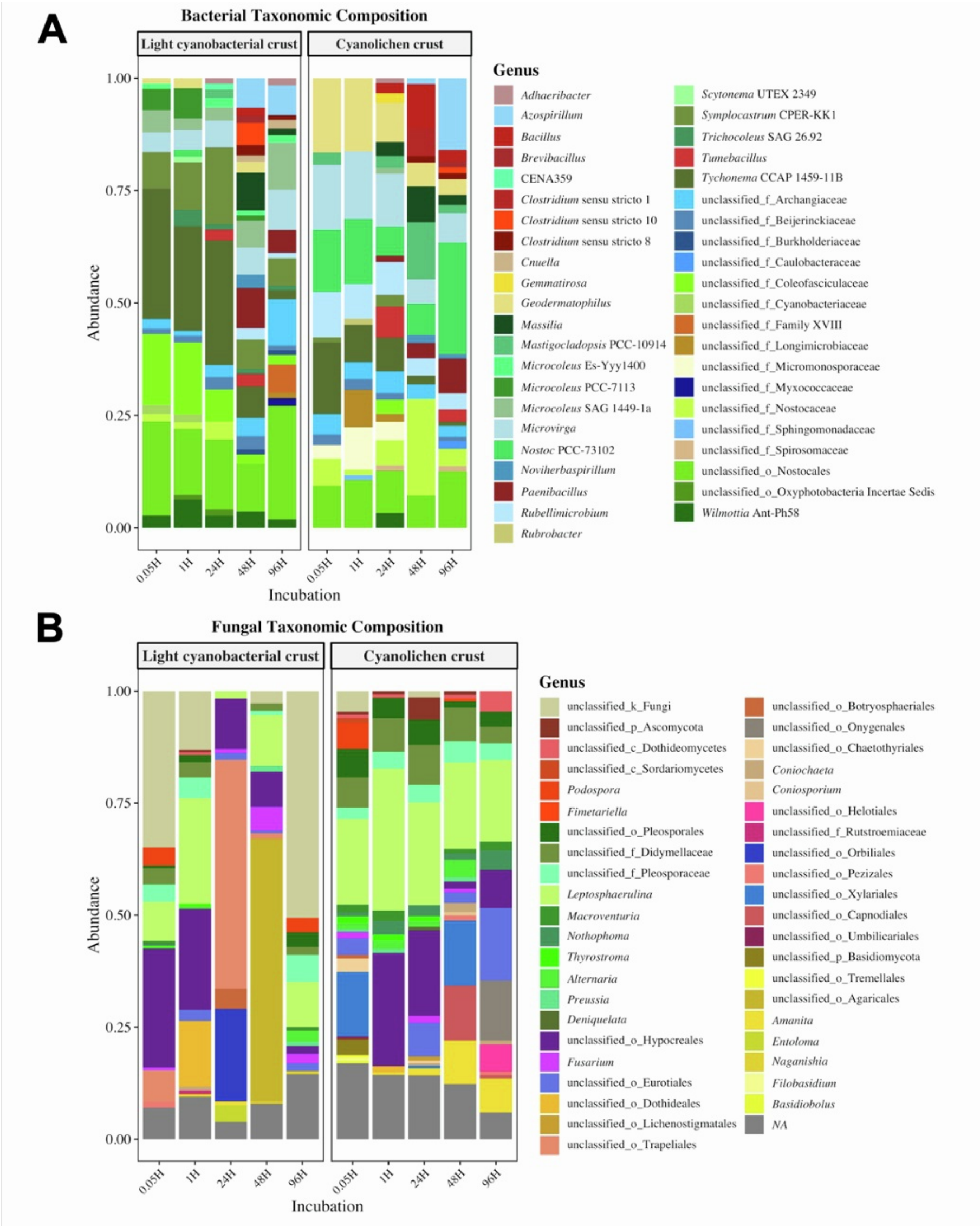

Figure S6. Taxonomic composition of Bacteria (A) and Fungi (B) at the genus level with relative abundance higher than 0.005 (0.5 percent). “others” represents a group of taxa which have an abundance < 0.5%.

Supplementary table

Table S1. Relative abundance of the dominant bacterial phyla in light cyanobacterial crusts and cyanolichens. Values range from 0 to 1, where 1 represents 100% relative abundance. Differences among incubation times were evaluated by one-way ANOVA followed by Tukey's post hoc test. Different lowercase letters within the same row indicate significant differences among incubation times (P < 0.05).

Crust type	Phylum	Abundance					ANOVA
		0.05 H	1 H	24 H	48 H	96 H	
LCC	Cyanobacteria	0.793a	0.758a	0.662b	0.359c	0.440d	F = 121.7, *p* = 2.92e-13
	Actinobacteria	0.043ab	0.053a	0.038b	0.044ab	0.022c	F = 15.04, *p* = 1.06e-05
	Firmicutes	0.001a	0.005a	0.0199ab	0.159c	0.117bc	F = 9.77, *p* = 0.000182
	Proteobacteria	0.114b	0.131ab	0.181a	0.339c	0.324c	F = 49.3, *p* = 8.95e-10
CLC	Cyanobacteria	0.352ab	0.249b	0.325ab	0.399a	0.371a	F = 1.244, *p* = 0.324
	Actinobacteria	0.181a	0.219a	0.113b	0.078bc	0.045c	F = 21.36, *p* = 5.49e-07
	Firmicutes	0.002a	0.003 a	0.081 ab	0.175 b	0.167 b	F = 11.8, *p* = 4.38e-05
	Proteobacteria	0.341a	0.331a	0.331a	0.274a	0.348a	F = 1.402, *p* = 0.269

Table S2. The linkages among the vertices in the network. LCC and CLC denote light cyanobacterial crust and cyanolichen, respectively. The numbers 1, 2, and 3 represent the initial, intermediate, and late response clusters.

**Connectivity terms**	**Metric**	**Number of linkages of whole communities in the network**					
		**LCC_1**	**LCC_2**	**LCC_3**	**CLC_1**	**CLC_2**	**CLC_3**
Fungi-Bacteria	Total	793	66	268	365	729	18
	Positive	387	38	218	246	421	15
	Negative	406	28	50	119	308	3
Fungi-Metabolite	Total	61	0	18	85	17	2
	Positive	29	0	13	60	9	1
	Negative	32	0	5	25	8	1
Bacteria-Metabolite	Total	222	112	25	53	46	2
	Positive	116	70	15	26	24	2
	Negative	106	42	10	27	22	0

Table S3. Lists of the putative keystone species and exometabolites in light cyanobacterial crust

Cluster	Topological roles	Module	ASV	Kingdom	Phylum	Order	Species
LCC 1	Connector	5	asv2201	Bacteria	Cyanobacteria	Nostocales	uncultured
	Module hub	1	Urocanic acid	Metabolite	Metabolite	Metabolite	Urocanic acid
	Module hub	10	Trigonelline	Metabolite	Metabolite	Metabolite	Trigonelline
	Module hub	2	asv286	Bacteria	Actinobacteria	Frankiales	uncultured
	Module hub	15	asv1483	Bacteria	Bacteroidetes	Cytophagales	uncultured
	Module hub	8	asv3454	Bacteria	Cyanobacteria	Nostocales	unidentified
	Module hub	21	asv603	Bacteria	Chloroflexi	Kallotenuales	unidentified
	Module hub	4	asv662	Bacteria	Gemmatimonadetes	Longimicrobiales	uncultured
	Module hub	16	asv9619	Bacteria	Cyanobacteria	Nostocales	*Scytonema* UTEX 2349
	Module hub	6	asv105	Bacteria	Proteobacteria	Rhodobacterales	*Rubellimicrobium* sp.
	Module hub	1	asv372	Bacteria	Proteobacteria	Rhizobiales	*Microvirga* sp.
	Module hub	4	asv414	Bacteria	Chloroflexi	Kallotenuales	uncultured
	Module hub	11	asv1146	Bacteria	Gemmatimonadetes	Longimicrobiales	*Longimicrobium* sp.
	Module hub	14	ASV47	Fungi	Ascomycota	Pleosporales	*Alternaria* sp.
	Module hub	3	ASV429	Fungi	Ascomycota	Umbilicariales	unidentified
	Module hub	3	ASV40	Fungi	N/A	N/A	unidentified
	Module hub	3	ASV320	Fungi	Ascomycota	Helotiales	unidentified
	Module hub	9	ASV30	Fungi	Ascomycota	Hypocreales	unidentified
	Module hub	5	ASV275	Fungi	Blastocladiomycota	Blastocladiales	unidentified
	Module hub	3	ASV228	Fungi	Ascomycota	Tubeufiales	unidentified
	Module hub	5	ASV176	Fungi	Ascomycota	Pleosporales	*Coniothyrium telephii*
	Module hub	3	ASV10	Fungi	Ascomycota	Hypocreales	*Fusarium* sp.
	Module hub	7	ASV1	Fungi	N/A	N/A	unidentified
LCC 2	Connector	2	asv240	Bacteria	Proteobacteria	Betaproteobacteriales	unidentified
	Connector	2	asv3205	Bacteria	Firmicutes	Bacillales	*Paenibacillus* sp.
	Module hub	1	5-Aminovaleric acid	Metabolite	Metabolite	Metabolite	5-Aminovaleric acid
	Network hub	3	3-Ureidopropionic acid	Metabolite	Metabolite	Metabolite	3-Ureidopropionic acid

LCC 3	Module hubs	7	asv13	Bacteria	Cyanobacteria	Nostocales	*Mastigocladopsis* PCC-10914
	Module hubs	3	asv716	Bacteria	Firmicutes	Bacillales	Paenibacillus sp. YN15
	Module hubs	6	asv23	Bacteria	Cyanobacteria	Nostocales	uncultured
	Module hubs	3	asv40	Bacteria	Cyanobacteria	Nostocales	uncultured
	Module hubs	7	asv391	Bacteria	Proteobacteria	Rhodobacterales	*Rubellimicrobium*
	Module hubs	3	asv8534	Bacteria	Firmicutes	Clostridiales	*Ruminiclostridium* 1
	Module hubs	2	ASV380	Fungi	Basidiomycota	Filobasidiales	*Filobasidium magnum*
	Module hubs	1	ASV1423	Fungi	Ascomycota	Pleosporales	unidentified

Table S4. Lists of the putative keystone species and exometabolites in cyanolichen crust

Cluster	Topological roles	Module	ASV	Kingdom	Phylum	Order	Species
CLC 1	Module hub	5	asv301	Bacteria	Bacteroidetes	Cytophagales	Adhaeribacter
	Module hub	13	asv1645	Bacteria	Gemmatimonadetes	Longimicrobiales	unidentified
	Module hub	16	asv408	Bacteria	Proteobacteria	Rhizobiales	metagenome
	Module hub	9	asv932	Bacteria	Gemmatimonadetes	Longimicrobiales	uncultured
	Module hub	12	asv78	Bacteria	Proteobacteria	Sphingomonadales	unidentified
	Module hub	17	asv1576	Bacteria	Proteobacteria	Acetobacterales	*Roseomonas* sp.
	Module hub	9	asv1009	Bacteria	Proteobacteria	Myxococcales	*Phaselicystis flava*
	Module hub	3	asv939	Bacteria	Cyanobacteria	Nostocales	uncultured
	Module hub	3	Carnitine	Metabolite	Metabolite	Metabolite	Carnitine
	Module hub	2	Pipecolic acid	Metabolite	Metabolite	Metabolite	Pipecolic acid
	Module hub	8	Serine	Metabolite	Metabolite	Metabolite	Serine
CLC 2	Module hub	15	ASV12	Fungi	Ascomycota	Pleosporales	unidentified
	Module hub	4	ASV49	Fungi	Ascomycota	Pleosporales	Thyrostroma cornicola
	Module hub	2	asv2073	Bacteria	Proteobacteria	Sphingomonadales	uncultured
	Module hub	3	asv492	Bacteria	Bacteroidetes	Cytophagales	uncultured
	Module hub	1	asv8145	Bacteria	Firmicutes	Bacillales	*Tumebacillus* sp.
	Module hub	6	asv638	Bacteria	Acidobacteria	Solibacterales	unidentified
	Module hub	7	asv1396	Bacteria	Firmicutes	Bacillales	*Bacillus* sp.
	Module hub	11	asv1388	Bacteria	Proteobacteria	Acetobacterales	*Craurococcus* sp.
	Module hub	3	asv576	Bacteria	Actinobacteria	Frankiales	unidentified
	Module hub	13	asv570	Bacteria	Cyanobacteria	Nostocales	uncultured
	Module hub	3	asv3919	Bacteria	Cyanobacteria	Nostocales	uncultured
	Connector	5	asv457	Bacteria	Proteobacteria	Rhizobiales	*Microvirga* sp.
	Connector	16	asv2	Bacteria	Cyanobacteria	Nostocales	*Nostoc* PCC-73102
	Module hub	11	asv128	Bacteria	Proteobacteria	Rhizobiales	*Salinarimonas* sp. BN140002
	Module hub	5	S-Adenosylhomocysteine	Metabolite	Metabolite	Metabolite	S-Adenosylhomocysteine
